# Maximized quantitative phosphoproteomics allows high confidence dissection of the DNA damage signaling network

**DOI:** 10.1038/s41598-020-74939-4

**Published:** 2020-10-22

**Authors:** Vitor Marcel Faca, Ethan J. Sanford, Jennifer Tieu, William Comstock, Shagun Gupta, Shannon Marshall, Haiyuan Yu, Marcus B. Smolka

**Affiliations:** 1 Department of Molecular Biology and Genetics, Weill Institute for Cell and Molecular Biology, Cornell University, Ithaca, NY 14853 USA; 2 Department of Biochemistry and Immunology and Cell-Based Therapy Center, Ribeirao Preto Medical School, University of Sao Paulo, Ribeirao Preto, SP 14049-900 Brazil; 3 Department of Computational Biology, Weill Institute for Cell and Molecular Biology, Cornell University, Ithaca, NY 14853 USA

**Keywords:** Kinases, Proteomics, DNA damage checkpoints, DNA damage response, Homologous recombination, DNA damage and repair, Post-translational modifications, Phosphorylation

## Abstract

The maintenance of genomic stability relies on DNA damage sensor kinases that detect DNA lesions and phosphorylate an extensive network of substrates. The Mec1/ATR kinase is one of the primary sensor kinases responsible for orchestrating DNA damage responses. Despite the importance of Mec1/ATR, the current network of its identified substrates remains incomplete due, in part, to limitations in mass spectrometry-based quantitative phosphoproteomics. Phosphoproteomics suffers from lack of redundancy and statistical power for generating high confidence datasets, since information about phosphopeptide identity, site-localization, and quantitation must often be gleaned from a single peptide-spectrum match (PSM). Here we carefully analyzed the isotope label swapping strategy for phosphoproteomics, using data consistency among reciprocal labeling experiments as a central filtering rule for maximizing phosphopeptide identification and quantitation. We demonstrate that the approach allows drastic reduction of false positive quantitations and identifications even from phosphopeptides with a low number of spectral matches. Application of this approach identifies new Mec1/ATR-dependent signaling events, expanding our understanding of the DNA damage signaling network. Overall, the proposed quantitative phosphoproteomic approach should be generally applicable for investigating kinase signaling networks with high confidence and depth.

## Introduction

Protein phosphorylation is of central importance in both normal physiology and pathological conditions. Phosphorylation-mediated switches regulated by protein kinases and protein phosphatases can affect protein structure and function, with consequences in enzymatic activity, protein localization, protein interactions and turnover^1–3^. The control circuits of the DNA damage response (DDR) are extensively regulated by phosphorylation, with the kinase Mec1 (human ATR) playing a major role in both activation of the DNA damage checkpoint as well as phosphorylation of substrates involved in a range of nuclear processes including DNA repair, DNA replication, and transcription^4–10^, To date a number of Mec1 substrates have been mapped by phosphoproteomics^11–13^. However, the current network of identified Mec1 substrates remains incomplete. Many DNA repair proteins are not highly expressed^14^, and represent a challenge for phosphoproteomic analyses of DNA damage signaling to achieve proper depth with high quality quantitative data. Improvements in global quantitative phosphoproteomic analyses are therefore necessary to comprehensively map the Mec1-dependent signaling network. Similar challenges exist for the study of other kinases and represent important barriers for progress in understanding kinase action in general.

Phosphoproteomics, the systematic and unbiased mapping of phosphorylation events, is achieved mainly using mass spectrometry (MS)-based approaches. Both instrumentation and bioinformatic tools applied for phosphopeptide identification have been continuously evolving^15–17^, culminating in large phosphoproteomic datasets in recent years^18–24^. In addition to in depth coverage of the phosphoproteome, comprehensive mapping of kinase-mediated signaling also requires quantitative analysis of each phosphopeptide or phosphorylation site to monitor its abundance in conditions of active kinase compared to conditions in which the kinase of interest is chemically and/or genetically ablated^11,25,26^. Various quantitative mass spectrometric approaches have been applied for the mapping of kinase signaling, including stable isotope labeling in cell culture (SILAC)^26–28^ and isobaric labelling strategies such as tandem mass tag (TMT)^29,30^. In a recent systematic comparison of quantitative phosphoproteomic strategies, SILAC was considered the most accurate, although TMT-based analyses yielded better coverage of the phosphoproteome^17^. SILAC is based on peptide precursor ion quantification to detect and quantify, in relative terms, the ratio between “heavy” and “light” isotopologues of amino acids (most commonly lysine and arginine) incorporated metabolically into cells^31,32^. Such an approach allows early mixing of labeled protein extracts in phosphoproteomic workflows to minimize technical variation.

Phosphoproteomics faces inherent issues for achieving identification and quantitation of phosphopeptides with high confidence. Different than proteomics, where the analysis of proteins is based on identification and quantification of multiple redundant representative peptides for a given protein, phosphoproteomics relies on phosphopeptides that are often unique (non-redundant) species represented by one or a few peptide spectral matches (PSMs) in the dataset. The lack of multiple redundant events for informing identification, quantification and phospho-site localization hobbles the acquisition of high-quality data due to the low numbers of PSMs per phosphopeptide^33^. The ability of acquiring high quality identification and quantification data is further complicated by the fact that many key phosphopeptides of biological interest are present at very low levels in the pool of phosphopeptides enriched from whole cell lysates. Even in cases when identification of a phosphopeptide based on one or two PSMs is successful, the associated quantitative information can suffer from signal interference derived from sample complexity and other intrinsic technical noise^34–37^. As a result, a significant part of the generated phosphoproteomic data is not suited for reliable quantitative analysis and biological inference, representing one of the major bottlenecks in large-scale quantitative phosphoproteomic analysis of kinase-mediated signaling.

Here we report a phosphoproteomic approach for increasing reliability in phosphopeptide identification and quantification, while minimizing loss of data from phosphopeptides with low PSM counts. The approach builds on the established concept of SILAC labeling swap, relying on quantitation consistency among reversed isotopically labeled samples as a central filtering step for removing false positive identifications and erroneous quantifications^31,38,39^. While isotopic label swapping has been a common practice in SILAC-based experiments^38–42^, its contribution to the reduction of false positive identifications and quantitations has not been systematically characterized, especially for cases of phosphopeptides with low PSM counts. By performing an in-depth analysis of label swap phosphoproteomics we monitor experimental error or biological variation in phosphopeptide quantitation and propose an approach for drastic reduction of false positive identifications and quantitation. The reported approach balances both sensitivity and specificity to detect phosphorylation changes with high confidence, even in the case of phosphopeptides with low PSM counts. Overall, the simple approach presented here enhances the reliability of quantitative phosphoproteomics in biological interrogations of kinase-mediated signaling networks.

## Results

### Error and variation in SILAC-based phosphopeptide quantitation is unidirectionally biased

**Figure 1:**
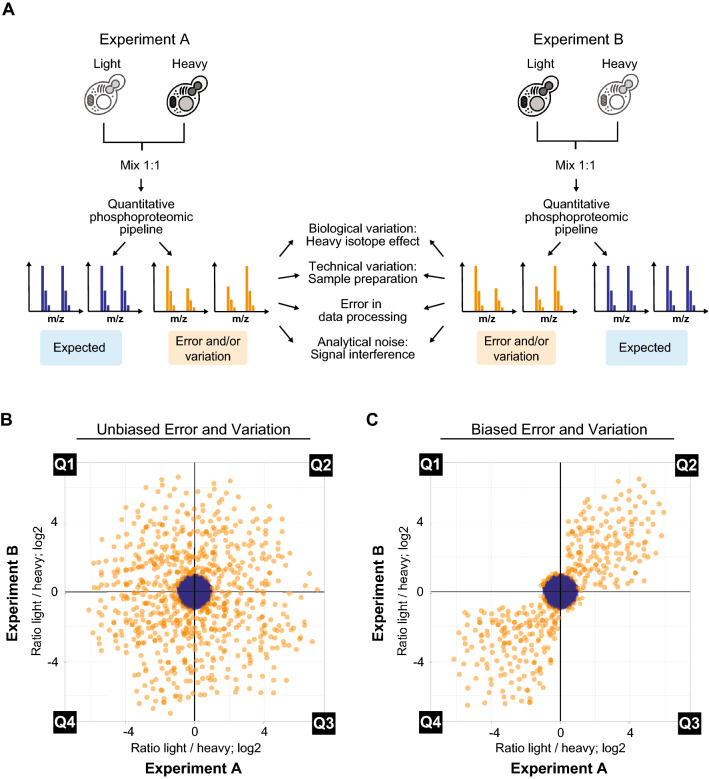
Modeling outcomes of SILAC reciprocal labeling as a means to reduce technical error and/or variation (EV). (**A**) Workflow showing reciprocal labeling scheme with a forward experiment (Experiment A, left) and a reverse experiment (Experiment B, right) with anticipated outcomes and proposed causes of EVs (middle). (**B**) Anticipated distribution of false positives in a comparison of two identical samples if error and variation occurred randomly and independent of isotopic labeling. (**C**) Anticipated distribution of false positives in a comparison of two identical samples if error and variation were unidirectionally biased (i.e. similar ratio in both a forward and reverse experiment).

We set out to develop an approach to maximize confidence in quantitative data from phosphoproteomic experiments. We postulated that SILAC-based quantitation might be particularly well suited for separating meaningful biological changes from: (1) aberrant quantitation during data processing (herein referred as “Error”), and/or (2) changes in phosphopeptide abundance unintentionally introduced during sample handling (herein referred as “Variation”). If both Error and/or Variation (EV) are mostly associated with artifacts that are independent of true biological differences in the cell lines or drug treatment conditions being compared, phosphoproteomic analysis should reveal a unidirectional bias in the generated ratios of data points reflecting EVs (Fig. 1A–C). We further reasoned that a strong bias in EVs would enable their systematic exclusion from large-scale phosphoproteomic datasets and, in principle, enable the generation of high confidence quantitative data even from phosphopeptides with only one PSM detected in each reciprocal, labeling swap SILAC experiment.

**Figure 2:**
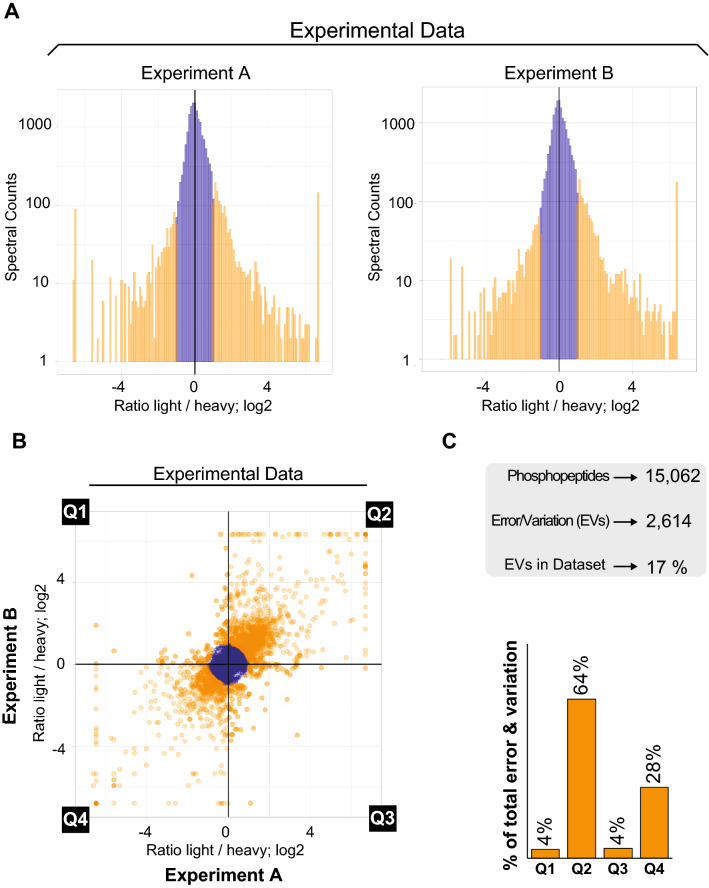
Reciprocal labeling in an isogenic yeast cell line reveals extensive error and variation that is unidirectionally biased. (**A**) Histograms for SILAC ratios of two independent phosphoproteome experiments comparing isogenic wild-type *S. cerevisiae* as depicted in Fig. 1A. EVs are colored in orange. (**B**) Scatterplot comparing experimental data from the two SILAC experiments shown in (**A**). EVs are colored in orange. (**C**) Histogram showing unidirectional bias of error and variation toward quadrants 2 and 4 in plot from (**B**).

To test this idea, we mixed equal amounts of protein extracts from budding yeast grown in light (^12^C^14^N arginine and lysine) or heavy (^13^C^15^N arginine and lysine) SILAC media and subjected lysates to a quantitative phosphoproteomic and data analysis pipeline outlined in Fig. 1 and detailed in Supplemental Figure S1. An independent biological replicate was performed to mimic a reciprocal, labeling swap experiment. As shown in Fig. 1A, data points with a SILAC ratio not reflecting the expected 1:1 ratio (simulated within a 33% coefficient of variation, or approximately a twofold change), were considered to reflect methodological error and/or variation. Comparison of experiments A and B (control and reciprocal label swap) should reveal if the error and/or variation exhibit any biased distribution in a quantitative plot (Fig. 1B,C). As shown in Fig. 2A (see Supplementary Table S1 for detailed dataset), separate experiments revealed thousands of data points outside a simulated range of 33% coefficient of variation (indicated in yellow). We reasoned that these points reflect EVs in the experiment. Notably, comparison of the ratio of each phosphopeptide in experiments A and B revealed a clear bias in EV distribution toward quadrants Q2 and Q4 (Fig. 2B,C) such that 92% of all EVs fell within these quadrants. Notably, EVs accounted for 17% of all phosphopeptides present in our dataset when considering phosphopeptides with 1 PSM in each experiment, underscoring the importance of their exclusion. Data points in Q2 and Q4 represent phosphopeptides whose SILAC ratios did not revert in the reciprocal experiment. Overall, these results reveal that the use of a SILAC labeling swap in phosphoproteomic experiments allows efficient detection of intrinsic EV in the dataset, which may be used for achieving high confidence quantitative analysis, even for phosphopeptides represented by a low number of PSMs. This ability to filter signal from noise, even when PSM numbers are low, is crucial for phosphoproteomic experiments which often rely on difficult-to-detect phosphopeptides. In fact, approximately a third of the data points in the correlation plot shown in Fig. 2B reflect phosphopeptides with only one PSM in one of the experiments (see Supplementary Table S1).

### Data filtering approaches for reducing error and variation

**Figure 3:**
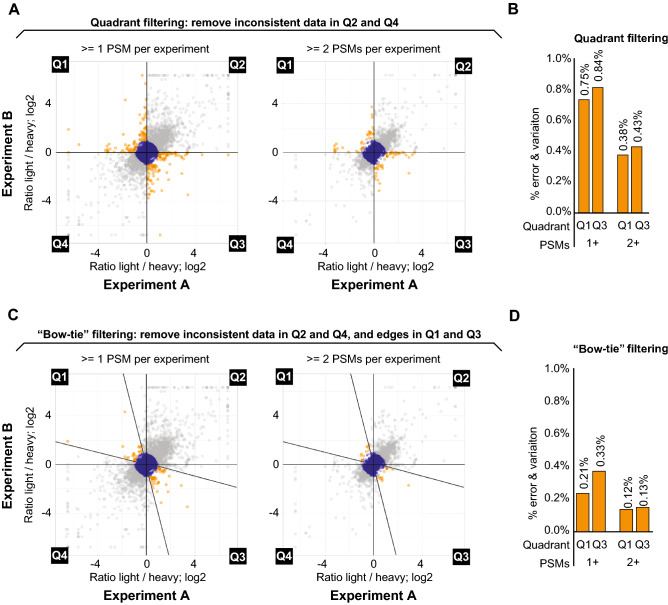
Data filtering based on quantitation consistency drastically reduces error and variation. (**A**) Scatterplot from Fig. 2B. indicating the “Quadrant” filtering scheme (gray data points removed in Q2 and Q4) and additional filtering based on the requirement for at least 2 PSMs per experiment for each data point (plot on the right). (**B**) Histogram showing EVs in Q1 and Q3 (orange data points) from A as a percentage of total dataset using either 1 PSM or 2 PSM filtering. (**C**) Scatterplot from Fig. 2B. indicating the “Bow-tie” filtering scheme (gray data points removed) and additional filtering based on the requirement for at least 2 PSMs per experiment for each data point (plot on the right). For Bow-tie filtering, in addition to removing EVs in Q2 and Q4, data points in Q1 and Q3 were required to be within an interval of correlation correspondent to fourfold of the log2 scale. (**D**) Histogram showing highlighted points in quadrants 1 and 3 from (**C**) as a percentage of total dataset using either 1 PSM or 2 PSM filtering.

To apply data filtering approaches for efficiently eliminating EVs while minimizing loss of data, we evaluated the effects of imposing thresholds on the minimal number of observations (PSMs) required for each phosphorylation site identified. While each phosphosite requires at least 2 observations (1 in each of the reciprocal experiments) to be shown in the correlation plot, increasing the requirement for 2 or more observations in each experiment decreased the proportion of EVs in relation to the entire dataset (Fig. 3A). Considering specifically data points present in Q1 and Q3, where inverse correlation is expected between phosphopeptide ratios in reciprocal experiments, we find that the proportion of EVs is about 1.5% when considering 1 or more PSM in each experiment. This EV proportion is reduced by approximately half, to 0.8% of the data points, when a minimum of 2 observations is required in each experiment (Fig. 3B). However, this additional requirement also decreased sensitivity, reducing the total number of data points from 15,062 to 10,439 (Supplementary Table S1).

During our EV analyses, we noticed a clear prevalence of data points close to the X-axis and Y-axis in Q1 and Q3 (Fig. 3A,C), revealing data points with a deviated ratio in only one of the experiments. By employing a simple “quadrant filtering” approach, whereby points in Q2 and Q4 are excluded, and points in Q1 and Q3 are kept, we cannot exclude highly variable phosphopeptide measurements that are also likely the result of error and/or variation (Fig. 3C). To circumvent this issue and more efficiently remove EVs for improved data quality, we designed an alternative filtering approach where data points in Q1 and Q3 were required to be within an interval of correlation correspondent to fourfold of the log2 scale (hereafter referred to as “Bow-tie filtering”) (Fig. 3C). As shown in Figs. 3C,D, the use of Bow-tie filtering, even where peptides with 1 PSM in each experiment were included, reduced the proportion of EVs to 0.54% of the dataset. When Bow-tie filtering was combined with the threshold of at least 2 PSMs per experiment, the proportion of EVs again dropped by approximately half to 0.25% of the dataset. These results reveal that the ability to identify EVs in SILAC-based phosphoproteomic experiments allows the utilization of filtering strategies that drastically reduce error and variation in the dataset, therefore increasing the confidence in the data even when considering phosphopeptides represented by a single PSM per experiment.

### Eliminating error and variation in quantitation reduces decoy identifications

SILAC labeling with stable isotopes shifts the mass of parent ions and their fragments in both MS1 and MS2, respectively. We reasoned that this mass shift should enable more efficient exclusion of false positive identifications in the dataset, since a misidentification would need to occur in both reciprocal experiments and be consistent between two parental ions with different m/z. To give a more detailed example, a false identification in a ^12^C^14^N (light) sample with a high light/heavy ratio, should not be reciprocally identified in the ^13^C^15^N (heavy) form, or if identified in the light form in the reciprocal experiment, it should not display an inverted low light/heavy ratio. If most of these cases reflect intrinsic experimental artefacts consistently present over biological replicates, independently of SILAC labeling swap, these false identifications should be prevalent in quadrants Q2 and Q4, because the peptide in question would be very unlikely misidentified in a reciprocal experiment due to its having a different m/z and/or display an inverted ratio. In such a context, consistency in quantitation over two or more biological replicates of label swapped experiments could be used as a parameter for efficiently excluding false identifications from final datasets, especially in the region of data points with high fold changes containing most of the key data that would be used for biological inference, such as for the identification of kinase substrates.

**Figure 4:**
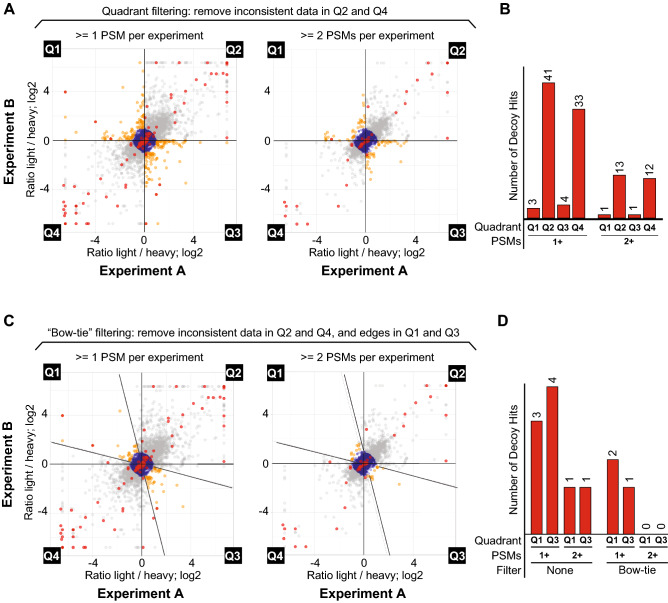
“Bow-Tie” approach efficiently reduces decoy peptide identifications. (**A**) Scatterplot from Fig. 2B. indicating hits from a decoy database. Decoy peptides are displayed in red. As in Fig. 3, unfiltered EVs in Q1 and Q3 are displayed in orange. (**B**) Histogram displaying number of decoy peptide hits in Q1 and Q3 with quadrant filtering applied. (**C**) Scatterplot from Fig. 2B. indicating hits from a decoy database and employment of Bow-tie filtering. Decoy peptides are displayed in red. As in Fig. 3, unfiltered EVs in Q1 and Q3 after Bow-tie filtering are displayed in orange. (**D**) Histogram displaying number of decoy peptide hits in Q1 and Q3 with Bow-tie filtering applied.

To test if performing a reciprocal labeling experiment indeed reduces false-positive identification and quantification, we estimated the error rate of phosphopeptide identifications by monitoring the distribution of reversed decoy hits from the list of phosphopeptide identifications that passed our basal quality criteria (PeptideProphet > 0.9 and < 20 ppm precursor ion error). As shown in Fig. 4A,B, decoy hits display a clear distribution bias towards quadrants Q2 and Q4, congruent with our rationale that false identifications are mostly unidirectional in quantitation and likely reflect artefacts that are extremely unlikely to occur in two reciprocal experiments, independently. Of all decoy hits in the unfiltered dataset, more than half (81 out of 130) were found to display ratios outside the twofold change range (Fig. 4A,B; Table 1). Notably, we were able to remove all decoy hits from Q1 and Q3 (regions expected to contain key data for biological inference of true changes in phosphorylation events) using the Bow-tie filtering strategy in combination with a threshold of at least 2 PSMs per experiment (Fig. 4C,D; Table 1). Even when phosphopeptides reflected by 1 PSM per experiment were allowed in the dataset, the number of decoy hits in Q1 or Q3 remained low (2 hits) (Fig. 4D). We also tested a stringent filter for phosphorylation site localization (PTMProphet score equal or above 0.9), which further reduced EVs in Q1 and Q3 to 0.36% without drastically reducing the overall coverage of the dataset (Table 1). These findings highlight the usefulness of our approach, which hinges on conducting a reciprocal SILAC experiment to improve confidence in both identification and quantitation in phosphoproteomic studies. Importantly, the described approach results in minor loss of valuable data content from low abundance phosphopeptides represented by only one PSM in each of the two reciprocal experiments.

**Table 1:** Summary of phosphoproteomic data from Figs. 2, 3 and 4, comparing Quadrant to Bow-tie filtering, 1 PSM cutoff to 2 PSM cutoff, and filter for PTMPROPHET score ≥ 0.9.

Minimal # of PSMs per experiment	Total phospho sites matched*	Identification		Quantitation		Data filter	Phospho-sites with consistent quantitation (center)	Identification		Quantitation	
		Total # of decoys hits	% of decoy hits	Total # of error and variation	% of error and variations			Persisting decoy hits in Q1 + Q3	% of decoy hits post-filtering	Persisting error and variation in Q1 + Q3	% of error and variation post-filtering
1	15,062	130	0.86	2614	17.35	Quadrant	12,448	7	0.06	198	1.59
1	15,062	130	0.86	2614	17.35	Bow-tie	12,448	3	0.02	67	0.54
2	10,439	46	0.44	1263	12.10	Quadrant	9176	2	0.02	75	0.82
2	10,439	46	0.44	1263	12.10	Bow-tie	9176	0	0.00	23	0.25
**Data filter: PTMPPROPHET score **≥** 0.9**											
1	13,391	118	0.88	2232	16.67	Quadrant	11,159	8	0.07	143	1.28
1	13,391	118	0.88	2232	16.67	Bow-tie	11,159	4	0.04	40	0.36
2	9771	41	0.42	1149	11.76	Quadrant	8622	2	0.02	61	0.71
2	9771	41	0.42	1149	11.76	Bow-tie	8622	0	0.00	16	0.19

### High confidence dissection of the Mec1-dependent signaling network

**Figure 5:**
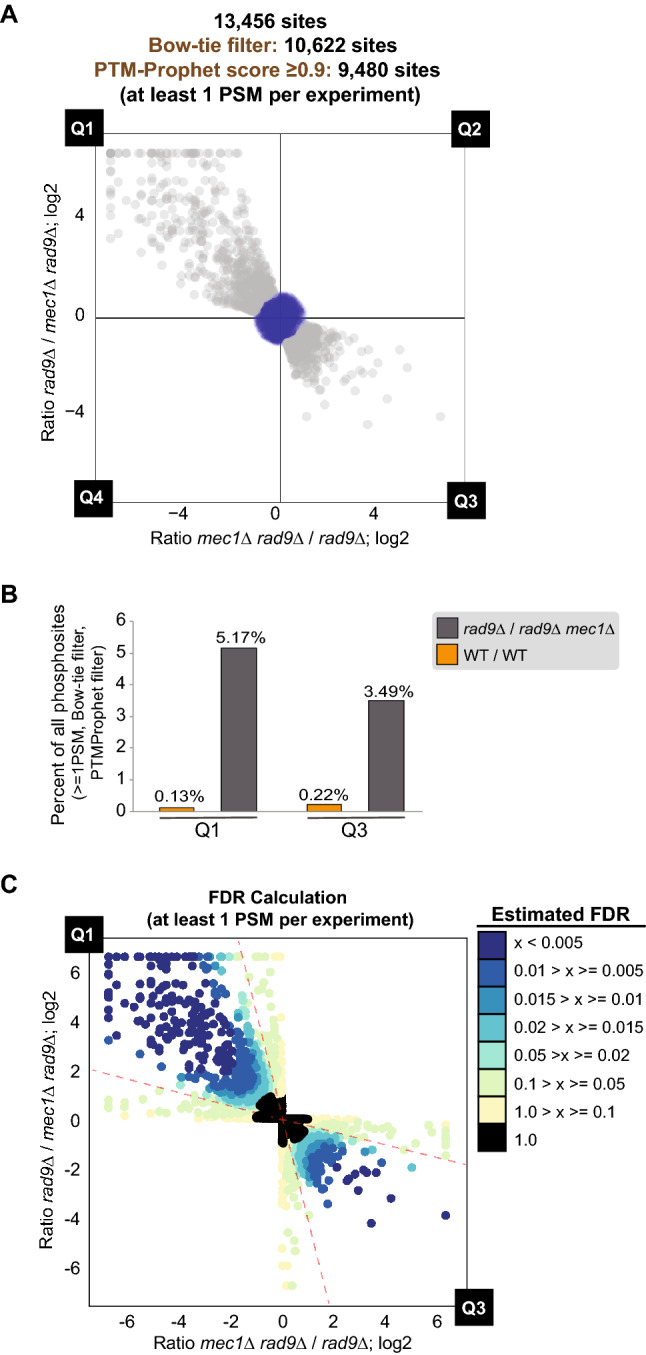
Quantitative phosphoproteomic analysis of Mec1-dependent signaling. (**A**) Scatterplot (with Bow-tie filter applied and PTMProphet score ≥ 0.9; only data points within Bow-tie filter displayed) of forward and reciprocal SILAC experiment comparing phosphoproteome of *rad9*Δ cells to phosphoproteome of *rad9*Δ *mec1*Δ cells. Cells were treated with 0.02% MMS for 2hrs. (**B**) Histogram depicting distribution of phosphorylation sites in Q1 and Q3 compared to control experiments. (**C**) Estimation of false discovery rate (FDR) in quantitative analysis for experiment in 5A. FDR for quadrants 1 and 3 is estimated based on error and variation in wild-type control experiment (Fig. 2). See “Materials and methods” for more details.

Mec1, the *Saccharomyces cerevisiae* ortholog of mammalian ATR, is a phosphoinositide 3-kinase-related kinase (PIKK) kinase that is a key mediator of DNA damage responses^43–45^. We have previously used quantitative phosphoproteomics comparing WT and *mec1Δ* cells to uncover phosphorylation events dependent on Mec1^11^. Here we applied our optimized quantitative phosphoproteomic approach to the study of Mec1 in order to benchmark our bow-tie approach and expand the Mec1-dependent signaling network. We carried out the experiments in cells treated with the DNA alkylating agent MMS (methyl methanesulfonate) and lacking the checkpoint adaptor Rad9 to minimize indirect downstream phosphorylation and preferentially reveal direct Mec1 substrates^6,46^. Overall phosphoproteome coverage was similar to the control experiment, with approximately 20,000 phosphopeptide identifications for each SILAC reciprocal experiment. Upon application of our most relaxed filtering scheme, which considers phosphopeptides with 1 or more PSM in each experiment and a PeptideProphet score of 0.9 or greater, a total of 13,456 unique phosphosites from 2778 different proteins were identified. In order to ensure confidence in phosphopeptide localization, we applied a PTMProphet score filter of greater than or equal to 0.9, somewhat reducing the total number of unique phosphopeptides identified in the Mec1 experiment to 11,950. The list of all phosphosites identified and quantified in our experiment is supplied in Supplemental Table S2. As shown in Fig. 5A, Q1 after Bow-tie filtering contained a large number of phosphosites consistently downregulated in *rad9*Δ cells lacking Mec1 in both reciprocal SILAC experiments. The number of phosphosites in Q1 was approximately equal to the number of EVs in Q2 and Q4 (Supplemental Table S3), indicating that if experiments we performed using only one labeling scheme, many of these EVs excluded in our Bow-tie approach would have been erroneously called Mec1-dependent sites, obfuscating true biological effects of *MEC1* loss. Reassuringly, phosphopeptides in Q1 or Q3 (representing phosphorylation events lost or induced upon deletion of *MEC1*) were approximately eight times more prevalent than in the WT (1:1) control experiment (Fig. 5B). Our filtering strategy allows minimal loss of data while increasing stringency for identification and exclusion of false positives through SILAC label swapping. To systematically and quantitatively demonstrate that the set of Mec1-dependent phosphorylation events had a low rate of EVs, we sought to stratify the bow-tie filter into several bins of increasing fold-change and calculate the false discovery rate (FDR) for each bin. Mathematically, the FDR is equal to the number of points in a given bin in the control-experiment divided by the number of points in a given bin in the Mec1 experiment. For the purposes of this calculation, points in Q1 and Q3 were considered together (Supplemental Figures S2A,C). Expectedly, FDR decreased with increasing distance from the center and was further reduced depending on how close points fell to the line of symmetry (Supplemental Figures S2B,D). The majority of the data points in Q1 and Q3 encompassed by the bow-tie filter have a p value less than 0.05 (Fig. 5C), thus validating our bow-tie filtering approach as a means to improve data quality while allowing the inclusion of difficult-to-detect phosphorylation events.

**Figure 6:**
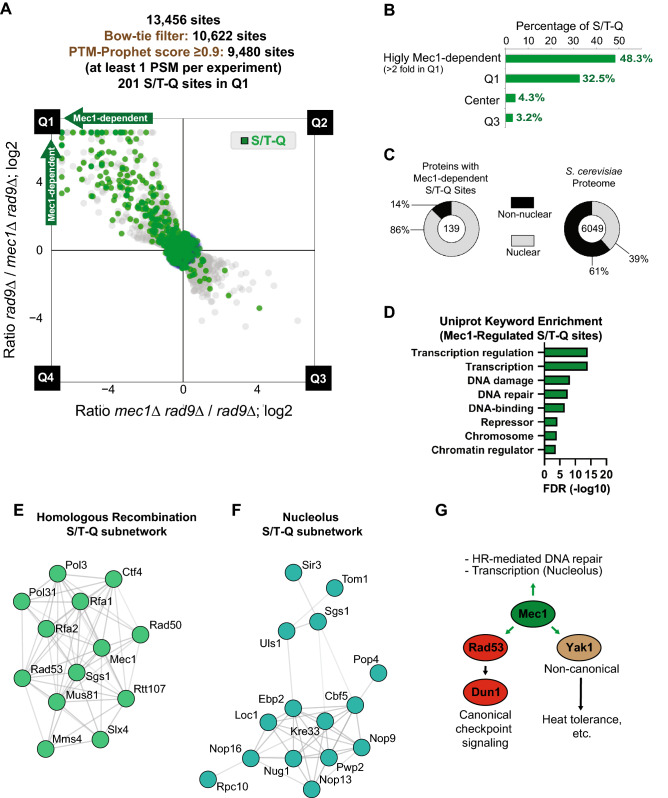
Expanding the Mec1 signaling network. (**A**) Scatterplot (with Bow-tie filter applied and PTMProphet score ≥ 0.9; only data points within Bow-tie filter displayed) of forward and reciprocal SILAC experiment comparing phosphoproteome of *rad9*Δ cells to phosphoproteome of *rad9*Δ *mec1*Δ cells. S/T-Q consensus motif is highlighted in green. Cells were treated with 0.02% MMS for 2 h. (**B**) Histogram of proportion of S/T-Q phospho-motif by quadrant in the Mec1 experiment from (**A**). (**C**) Pie chart showing proportion of nuclear proteins (GO Cellular Location^79,80^) in Mec1-dependent (log2 ratio > 1.0) S/T-Q sites from the Mec1 experiment in (**A**). (**D**) Uniprot keyword enrichment analysis performed on proteins containing Mec1-dependent S/T-Q phosphorylation from (**A**). (**E**) String analysis of proteins with Mec1-dependent phosphorylation in the S/T-Q consensus revealed a sub-network of proteins involved in DNA repair via homologous recombination (HR). Image adapted from https://string-db.org/. (**F**) String analysis of proteins with Mec1-dependent phosphorylation in the S/T-Q consensus revealed a sub-network of proteins related to the nucleolus. Image adapted from https://string-db.org/. (**G**) Simplified model depicting major processes directly regulated (green arrows) by Mec1. Results suggest that the Yak1 represents a novel kinase under direct control by Mec1.

The results of our experiment revealed an extensive network of Mec1-dependent phosphorylation events, many not published before and mostly phosphorylated at the preferential S/T-Q motif (Fig. 6A, green dots), which was overrepresented in Q1 (Fig. 6B). Whereas the S/T-Q motif represents only about 3% of the phospho-sites in the entire dataset, it represents 33% of the Q1 sites, and 49% of the group of highly Mec1-dependent sites (over twofold depletion in *rad9Δmec1Δ* cells). Besides S/T-Q sites, Q1 also contained a number of sites with the S/T-ψ (where ψ denotes the bulky hydrophobic residues F, I, L and V) phospho motif (Supplemental Table S3), which is associated with the downstream checkpoint kinase Rad53 that is activated by Mec1^47^. The occurrence of S/T-ψ phosphorylation in the absence of the major *RAD9*-dependent pathway of Rad53 activation likely reflects Rad53 activation via the Mrc1 adaptor^48^. Indeed, Mec1-dependent phosphorylation sites were detected in Rad53, several of which are known Rad53 autophosphorylation sites^49^ and indicate that this kinase is activated in *rad9Δ* cells expressing Mec1.

In total, our quantitative phosphoproteomic approach using Bow-tie filtering of inconsistent ratios resulted in the identification of 201 S/T-Q Mec1-dependent phosphosites, which at least triples the number of Mec1 targets identified compared to our previous screen^11^. Consistent with Mec1 being a nuclear kinase, these sites identified in our screen occurred largely on nuclear proteins (Fig. 6C). Gene enrichment analysis of all Mec1-regulated S/T-Q sites in Q1 (with a log2 ratio > 1 in *rad9*Δ cells relative to *mec1*Δ *rad9*Δ) was consistent with our previous study showing that the substrate repertoire of this kinase was enriched for nuclear proteins involved in DNA repair, chromatin dynamics, and transcription (Fig. 6D; Supplemental Table S4). String network analysis^50^ of the proteins with regulated S/T-Q sites revealed extensive Mec1-dependent phosphorylation of components of the homologous recombination machinery (Fig. 6E), including proteins such as Rad50 that act early in HR during the resection step^51,52^, as well as proteins that act later during HR to regulate the processing of joint molecules, such as Sgs1 and Mus81-Mms4^53–55^. Additionally, we found extensive Mec1-dependent phosphorylation of nucleolar proteins at the S/T-Q consensus, suggesting direct control of nucleolar processes by Mec1 (Fig. 6F). Analysis of Mec1-regulated sites containing a consensus motif that was not S/T-Q revealed that the scope of Mec1’s downstream signaling also largely encompassed proteins related to DNA damage, repair, and transcription, while also showing prevalence of cell-cycle, DNA replication and cytoplasmic proteins (Supplemental Figure S3A; Supplemental Table S5). Similar to the SQ consensus sites, the majority of the non-SQ sites were in nuclear proteins (Supplemental Figure S3B). String analysis of non-S/T-Q signaling events in the “cell cycle” node revealed non-canonical Mec1-dependent phosphorylation of the spindle assembly protein Mad3 and the condensin subunit Smc4 (Supplemental Figure S3C).

We also identified Mec1-dependent phosphorylation sites in the Dun1 kinase, which is known to function downstream of Mec1 and Rad53 in the canonical DNA damage checkpoint signaling pathway^56–58^. Interestingly, our SILAC-based filtering approach revealed a number of Mec1-dependent sites that did not contain the S/T-Q or S/T-ψ consensus, raising the possibility that Mec1 regulates the action of other kinases in addition to Rad53 and Dun1 in response to DNA damage. An example of a potentially new kinase targeted by Mec1 in our data is the DYRK-family kinase Yak1, which was phosphorylated in a Mec1-dependent manner in response to DNA damage on serine 663 (Supplemental Table S2; Fig. 6G). Both Yak1 and Mec1 have been reported to be important for acute heat shock resistance^59,60^, raising the possibility that Mec1 and Yak1 may be acting in the same stress-response pathway. Lastly, analysis of phosphorylation sites in Q3 revealed a likely up-regulation of the Tel1 kinase, a Mec1-related PI3K-like Kinase (PIKK) with roles in DNA double strand break (DSB) repair and telomere maintenance^61–63^. Q3 included phosphorylation of the telomere maintenance protein Rif1 at serine 1308, which was previously shown to be dependent on Tel1^64^. In fact, ATM/Tel1 signaling has been reported to be up-regulated in the absence of ATR/Mec1 in mammals^65–67^. Q3 also contained additional phosphorylation sites in proteins related to DNA double strand break (DSB) repair and telomere maintenance (Supplemental Figure S4). Notably, some of these phosphorylation sites were not present in the canonical S/T-Q motif (Supplemental Table S2), suggesting additional non-canonical Tel1-dependent phosphorylation and/or the involvement of additional kinases. Taken together, these findings highlight the efficacy of our optimized quantitative SILAC-based phosphoproteomic approach and Bow-tie filtering method in identifying and quantifying kinase-dependent signaling events at high depth and specificity, while minimizing false positives.

## Discussion

The field of phosphoproteomics has made significant strides toward improved phosphopeptide detection and quantitation since the seminal paper by Fenselau et al. which described the first application of FAB mass spectrometry for phosphopeptide characterization^68^. Throughput as well as robustness has increased, and modern instruments and workflows can routinely detect and quantitate thousands of phosphopeptides in a single run. Still, the intrinsic issue of lack of redundancy in data representation for each phosphopeptide remains, leading to lack of statistical power for generating high confidence quantitation and identification for large portions of the dataset, especially for low abundance phosphopeptides that often rely on single PSMs with noisy signals. This issue has been tackled predominantly by requiring higher numbers of PSMs per phosphopeptide, with the consequent trade-off of eliminating a substantial fraction of the dataset that may contain most of the biologically meaningful regulatory, and low abundant, events. This problem is especially salient for nuclear proteins involved in the DNA damage response which often exist at low levels in the cell^14^. In this work we present a workaround that allows for the efficient exclusion of technical noise and variation through the use of a reciprocal SILAC experiment, while allowing for the identification and quantitation of low abundance phosphopeptides. We leveraged the sensitivity of this pipeline by combining the proposed Bow-tie analysis with samples that had been pre-fractionated using HILIC chromatography. The result is a drastic expansion in coverage with concomitant reduction in error and technical variation in the overall quantitative data. This combination of high specificity, low-PSM phosphoproteomics with HILIC, which is particularly suited to phosphopeptide fractionation due to the hydrophilicity of the phosphate group^69^ revealed ~ 15,000 unique phosphopeptides in a short fractionation schema (15 fractions). Importantly, we demonstrate the utility of this approach by identifying new Mec1-dependent signaling events in *S. cerevisiae*.

Central to the Bow-tie filtering strategy presented in this study is the use of metabolic labelling with stable isotopes (SILAC) and the consequent shift in mass of parent and fragment ions of phosphopeptides. Such a large delta mass between phosphopeptides in reciprocal experiments forces a stringent requirement in which phosphopeptide identification with inverted fold change in each experiment should also exhibit proper delta mass shift. In addition to allowing efficient detection of EVs, this approach also led to a dramatic reduction in the number of decoy database peptide identifications in quadrants 1 and 3. This serves as definitive proof that reciprocal labeling reduces false-positive identification and associated quantitation. False-positives identifications are proposed to represent either artefacts, exogenous sample contaminants not represented in the searched database, or containing other types of modifications not considered in our search as variable modifications^70^. The Bow-tie approach applied to the mock dataset reduced false-positive hits to virtually zero, when Q1 and Q3 were considered and at a PeptideProphet score minimum score of 0.9, satisfying our needs for highly sensitive and comprehensive strategy to uncover phosphopeptides of low abundance and low PSM counts. A near-zero frequency of false-positive identifications appearing in Q1 and Q3 is essential to our SILAC-based approach, because peptide identification essentially serves as the most important gatekeeper of filtering meaningful biological data from technical noise.

The mass spectrometric data processing pipeline employed in this study relied on the trans-proteomic pipeline (TPP) suit of proteomic tools, including updated tools for peptide identification with Comet^71^ and scoring with PeptideProphet^72^. For SILAC quantitation, we used the Xpress precursor ion intensity quantitation tool^73^, and for phosphosite localization and scoring we used the newly described PTMProphet tool^74^. PTMProphet models the potential sites of phosphorylation independently of the spectrum identification provided by the search engine and calculates probabilities for each potential modification site. This feature allowed us to design additional steps in our R-based scripts for handling clustered S/T/Y residues, which is a common occurrence in phosphopeptides. Unambiguous, high-confidence phosphorylated S/T/Y residues with neighboring S/T/Y residues were kept separate; medium or low-confidence phosphorylated S/T/Y residues with adjacent S/T/Y residues were combined with their neighbors and considered in our subsequent analyses as a “cluster”.

The ability of our Bow-tie approach to separate biologically meaningful phosphorylation from technical noise is exemplified by the observed regulation in our Mec1 phospho-mapping dataset. In contrast to the control dataset, in which there were a small number of points in quadrants 1 and 3, there were a number of regulated sites in Q1 in our Mec1 dataset (and much less in Q3). S/T-Q consensus motif sites were overrepresented in Q1, indicating primary Mec1-dependent phosphorylation in response to DNA damage that was ablated in the absence of the *MEC1* gene. In addition to revealing many known Mec1 targets identified in other studies, which was our intention as a validation of our method, we revealed a number of previously unreported proteins with Mec1-dependent phosphorylation events, including a subset of nucleolar proteins. For example, we identified phosphorylation on serine 1007 (an S/T-Q site) of Kre33, a relatively understudied protein that promotes maturation of 18S rRNA^75,76^. Future work should be targeted toward understanding how Mec1 signaling contributes to nuclear homeostasis independently of its established roles in activation of the DNA damage checkpoint. One new kinase target of Mec1 present in our dataset is Yak1, which we found to be phosphorylated on Serine S663, near Yak1’s kinase domain. Yak1 is a member of the family of Ser/Thr protein kinases known as dual-specificity Tyr phosphorylation-regulated kinases (DYRKs). Yak1 has been described as a growth antagonist downstream of Ras/PKA pathway, phosphorylated by PKA and translocated to the nucleus upon nutrient deprivation^77^. Indeed, cells lacking *YAK1* are sensitive to acute heat stress^60^. Intriguingly, cells lacking *MEC1* are sensitive to proteotoxic and heat stress^59^. No previous reports have linked Yak1 to the DNA damage response or to Mec1, and we speculate that this could be a new point of crosstalk between DNA damage signaling and cellular stress responses.

In summary, here we report a simple, robust SILAC-based phosphoproteomic data analysis pipeline that allows for identification and quantitation of phosphopeptides with high confidence and coverage. The depth of the analyses allowed identification of a range of novel Mec1-dependent signaling events, including a potentially new mode of Mec1 signaling targeting the nucleolus. While this work highlights the utility of SILAC for high confidence and in depth quantitative phosphoproteomics, the same rationale could be applied to improve the quantitative analysis of other low-abundance post-translational modifications such as sumoylation, ubiquitylation, and acetylation.

## Materials and methods

### Yeast cell culture and manipulation

A list of yeast strains used in this study is found in Supplemental Table S6. The strain background for all yeast used was S288C. We performed whole ORF deletions of *MEC1* and *RAD9* using established PCR-based methods for amplifying resistance cassettes containing homology to the target gene. Gene manipulations were verified by PCR. Primers used for gene deletions are available upon request. Yeast were grown at 30 °C in synthetic SILAC media lacking arginine and lysine and supplemented with “light” lysine and arginine (^12^C and ^14^N) or supplemented with “heavy” lysine and arginine (l-lysine ^13^C_6_,^15^N_2_·HCl and l-arginine ^13^C_6_,^15^N_4_·HCl). Media was also supplemented with excess l-proline to prevent conversion of arginine to proline.

### Sample preparation for phosphoproteomic analysis

200–300 mL of yeast was grown in either “heavy” or “light” SILAC media to mid-log phase and treated as described in the figure legend and the text, depending on the experiment. Cells were pelleted at 1000×*g* and washed once with TE (10 mM Tris pH 8.0, 5 mM EDTA) buffer containing 1 mM PMSF. Cells were lysed by bead beating with 0.5 mm glass beads for 3 cycles of 10 min with 1-min rest time between cycles at 4 °C in lysis buffer (150 mM NaCl, 50 mM Tris pH 8.0, 5 mM EDTA, 0.2% Tergitol type NP40) supplemented with protease inhibitor cocktail (Pierce), 5 mM sodium fluoride and 10 mM β-glycerophosphate. 5–7 mg of each light and heavy labeled protein lysate was denatured and reduced with 1% SDS and 5 mM DTT at 42 °C, then alkylated with 25 mM iodoacetamide. Lysates (light and heavy) were mixed and precipitated with a cold solution of 50% acetone, 49.9% ethanol, 0.1% acetic acid. Post-precipitation protein pellet was then resuspended in 2 M urea and subsequently digested with TPCK-treated trypsin overnight at 37 °C. Phosphoenrichment was performed using a High-Select Fe-NTA phosphopeptide enrichment kit (ThermoFisher Scientific, cat# A32992) as described in the manufacturer’s instructions. Purified phosphopeptides were then dried in a SpeedVac and fractionated via HILIC chromatography as described below.

### HILIC fractionation

Dried phosphopeptide samples were reconstituted in 15 μL H_2_O, 10 μL 10% formic acid (v/v), and 60 μL HPLC-grade acetonitrile. 80 μL of the reconstituted sample was injected and fractionated by hydrophilic interaction liquid chromatography (HILIC) using a TSK gel Amide-80 column (2 mm × 150 mm, 5 μm; Tosoh Bioscience). Three solvents were used for the gradient: buffer A (90% acetonitrile), buffer B (75% acetonitrile and 0.005% trifluoroacetic acid), and buffer C (0.025% trifluoroacetic acid). A short gradient was used for the mock control and Mec1 experiments and consisted of 100% buffer A at time = 0 min; 88% of buffer B and 12% of buffer C at time = 5 min; 60% of buffer B and 40% of buffer C at time = 30 min; and 5% of buffer B and 95% of buffer C from time = 35 to 45 min in a flow of 150 µl/min. 30-s fractions were collected between 9 and 18 min. Individual fractions were dried in speedvac and submitted to LC–MS/MS analysis.

### Phosphoproteomics data acquisition

Individual phosphopeptide fractions were resuspended in 0.1% trifluoroacetic acid and subjected to LC–MS/MS analysis in an UltiMate 3000 RSLC nano chromatographic system coupled to a Q-Exactive HF mass spectrometer (Thermo Fisher Scientific). The chromatographic separation was carried out in 35-cm-long 100-µm inner diameter column packed in-house with 3 µm C_18_ reversed-phase resin (Reprosil Pur C18AQ 3 μm). Q-Exactive HF was operated in data-dependent mode with survey scans acquired in the Orbitrap mass analyzer over the range of 380–1800 m/z with a mass resolution of 60,000 (at m/z 200). MS/MS spectra was performed selecting the top 15 most abundant + 2, + 3 or + 4 ions and a with an precursor isolation window of 2.0 m/z. Selected ions were fragmented by Higher-energy Collisional Dissociation (HCD) with normalized collision energies of 28 and the mass spectra acquired in the Orbitrap mass analyzer with a mass resolution of 15,000 (at m/z 200), AGC target set to 1e^5^ and max injection time set to 120 ms. A dynamic exclusion window was set for 30 s.

### Phosphopeptide and phosphosite identification

The peptide identification and quantification pipeline relied on TPP tools^78^. The search engine used was Comet (v. 2019.01.1)^71^. Search parameters included semi-tryptic requirement, 20 ppm for the precursor match tolerance, differential mass modification of 8.0142 for lysine, 10.00827 for arginine, 79.966331 for phosphorylation of serine, threonine and tyrosine, 97.976896 for phosphorylation dehydration, and static mass modification of 57.021465 for alkylated cysteine residues. The protein sequence database was the SGD yeast supplemented with the decoy reversed sequences and common contaminants (downloaded in Aug 2019, 11,968 entries). Original ThermoScientific .raw files were converted to mzXML before the search with Comet. After searches, peptides were filtered and scored by the PeptideProphet algorithm^72^ using the following parameters: minimum probability of 0.9, minimum peptide length of 7 amino acid residues, accurate mass binning, restriction to + 2, + 3 and + 4 ion charge states and Phospho-Information enabled. After scoring and filtering, relative quantitation based on SILAC were obtained using Xpress and specific parameters were: mass tolerance of 0.005 daltons; minimum number of chromatogram points needed for quantitation = 1; number of isotopic peaks = 0. Phosphopeptides were then evaluated by PTMProphet^74^ in order to obtain accurate phosphosite localization score. The complete lists of identified, quantified, scored, and filtered phosphopeptides were further processed using a R-script developed in-house. The script separates phosphosites with high PTMProphet probability (> 0.9) from those with ambiguous localization containing 2 or more adjacent potentially phosphorylated residues, here denominated “clusters”. Separately, high confidence phosphosites and clustered phosphosites had their SILAC quantitation median calculated and additional R-scripts were used for combining, correlating, and plotting the data.

### Estimation of false discovery rate (FDR) in quantitative analysis

All points (from the mock and Mec1 experiment) belonging to quadrant 2 and 4 are removed along with all the points that have fold change (FC) of less than or equal to 2 and hence would lie in a circle with radius 1 (since the scale is log transformed FC). The points in quadrant 1 and quadrant 3 are combined in order to get symmetric parabolic bins. A choice of aperture is made by sampling this space using multiple parabolas rotated at 45° (~ 0.78 radians) with their vertex on the circle with radius 1. This is done to ensure accordance with the underlying assumption that the highest confidence points would lie far away from origin along the line of symmetry y = − x. False discovery rate (FDR) is calculated as the percentage of false positives given by the mock experiment to the false positives and true positives given by the Mec1 experiment that lie within each parabola. The false positives are indicated with red color and the true positives are indicated with green color in Supplementary Figure S1. The parabolic bins that gave FDR values closest to commonly used FDR values (5% and 2%) were retained and the bin aperture that gave a 2% FDR is then used to further sample the space by varying the vertex of the parabolas. The vertices were chosen so that the obtained FDR would be the first local minimum within an FDR range. This was done to ensure that the number of false positives are minimized, and the number of true positives are maximized.

### String analysis of S/T-Q and non-S/T-Q motif Mec1-dependent sites

A subset of phosphorylation sites (e.g. all S/T-Q sites in the experiment from Fig. 5 with a log2 ratio > 1) was selected and the list of gene names uploaded to https://string-db.org/. In cases where there were multiple sites under the same gene name entry, the gene name was used only once. Interaction networks were generated considering only high confidence interactions (score > 0.700). Next, the genes in the list corresponding to a specific biological process or pathway (e.g. nucleolus) were again uploaded to https://string-db.org/.

### Uniprot keyword enrichment analysis of S/T-Q and non-S/T-Q motif Mec1-dependent sites

A subset of phosphorylation sites (e.g. all S/T-Q sites in the experiment from Fig. 5 with a log2 ratio > 1) was selected and the list of gene names uploaded to https://string-db.org/. In cases where there were multiple sites under the same gene name entry, the gene name was used only once. Interaction networks were generated considering only high confidence interactions (score > 0.700). Next, the top 8-ranked Uniprot Keyword enrichment terms were exported along with the associated false discovery rate (FDR). For visualization, the FDR was log10-transformed. The terms “Nucleus” and “Phosphoprotein” were manually excluded from the figure because they represent processes that are too general to be informative.

## Data availability

Mass spectrometry data generated from this study has been deposited to the Massive database (https://massive.ucsd.edu). The control mock experiment data received the ID: MSV000084852, 10.25345/C58M3B, and ProteomeExchange ID: PXD017322. The Mec1 targets experiment data received the ID: MSV000084875, 10.25345/C56Q44, and ProteomeExchange ID: PXD017339.

## Supplementary information

Supplementary Figures

Supplementary Table 1

Supplementary Table 2

Supplementary Table 3

Supplementary Table 4

Supplementary Table 5

Supplementary Table 6
