# Supplementary material for: Maximized quantitative phosphoproteomics allows high confidence dissection of the DNA damage signaling network
Source: Sci Rep. 2020 Oct 22;10:18056. doi: 10.1038/s41598-020-74939-4 (PMC7582137; doi:10.1038/s41598-020-74939-4)
Supplement: Supplementary file 1 — Supplementary Figures [file 41598_2020_74939_MOESM1_ESM.pdf]

Supplementary information

Maximized Quantitative Phosphoproteomics Allows High Confidence Dissection of the DNA Damage Signaling Network

Vitor Marcel Faca[1,2,#], Ethan J. Sanford[1,#], Jennifer Tieu[1], William Comstock[1], Shagun Gupta[3], Shannon Marshall[1], Haiyuan Yu[3], and Marcus B. Smolka[1,*]

[1] Department of Molecular Biology and Genetics, Weill Institute for Cell and Molecular Biology, Cornell University, Ithaca, NY 14853, USA.

[2] Department of Biochemistry and Immunology and Cell-Based Therapy Center, Ribeirao Preto Medical School, University of Sao Paulo, Ribeirao Preto, SP, 14049-900, Brazil.

[3]Department of Computational Biology, Weill Institute for Cell and Molecular Biology, Cornell University, Ithaca, NY 14853, USA.

# These authors contributed equally to this work.

* Correspondence and requests for materials should be addressed to M.B.S. (email: mbs266@cornell.edu).

DATA PROCESSING PIPELINE

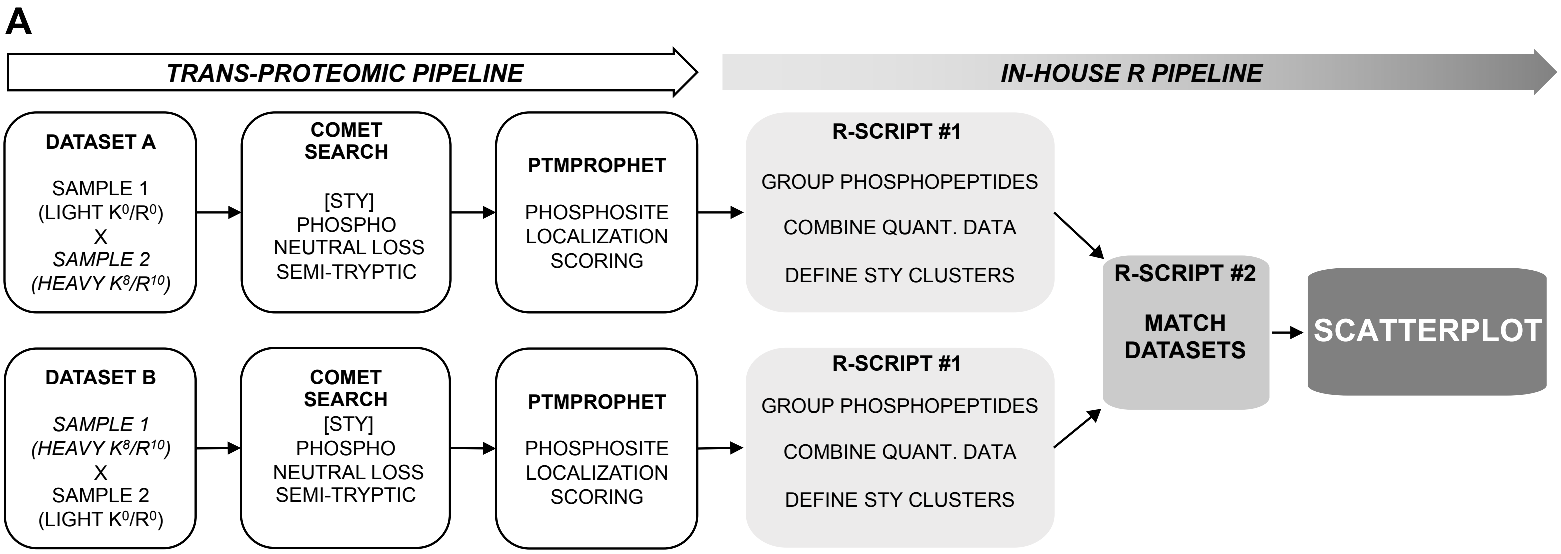

Supplemental Figure S1. A. Diagram representing the main steps of data processing for generating scatterplot of two datasets. The Trans-Proteomic Pipeline (TPP) was used as the core for major steps of phosphoeptide identification, quantification and phosphosite localization. Output tables (pep.xml) from TPP were further processed and results were organized using R-scripts described in the diagram.

SUPPLEMENTAL FIGURE 2
Corresponding to Figure 5

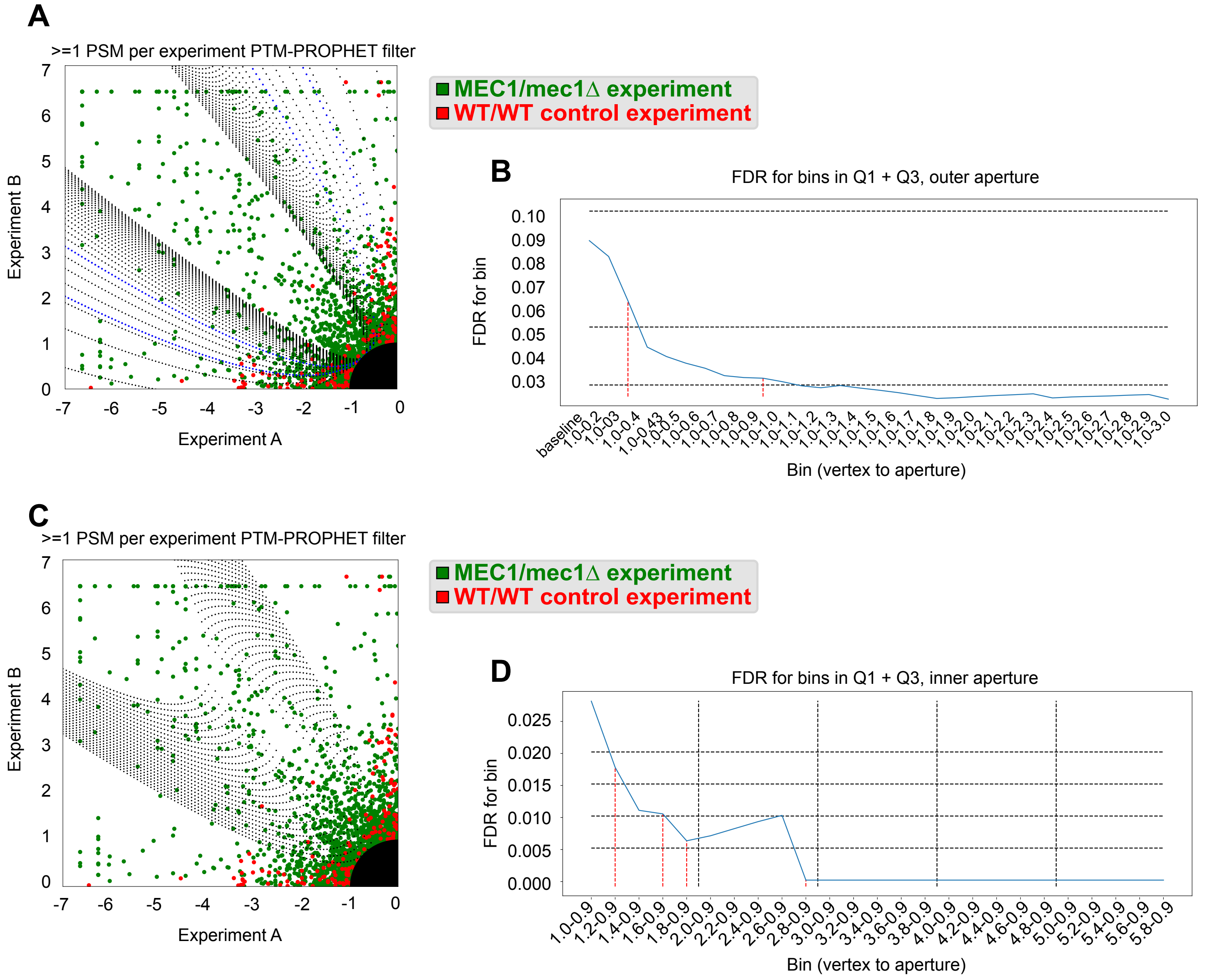

Supplemental Figure S2 (related to Figure 5).
A. Scatterplot of outer parabolas chosen for FDR filtering. Parabolas used in the analysis are highlighted in blue.
B. Graph indicating estimated FDR along parabolas with increasing aperture tightness.
C. Scatterplot of inner parabolas chosen for FDR filtering.
D. Graph indicating estimated FDR along parabolas with increasing aperture tightness and distance of vertex from origin.

SUPPLEMENTAL FIGURE 3
Corresponding to Figure 6

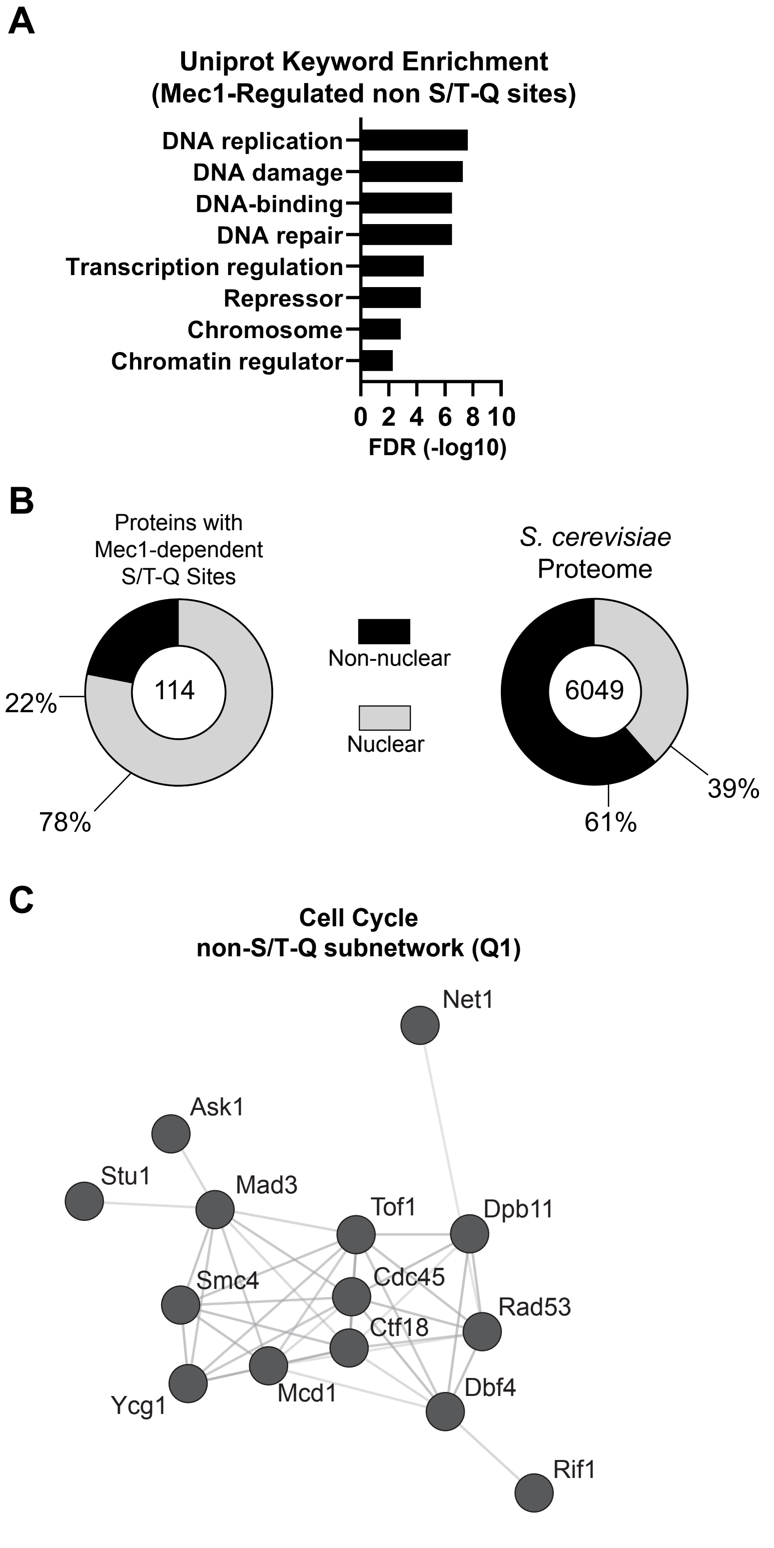

Supplemental Figure S3 (related to Figure 6).
A. Uniprot keyword enrichment analysis performed on all Mec1-dependent (Q1) non-S/T-Q consensus phosphorylation sites from Figure 6A.
B. Pie chart showing proportion of nuclear proteins (GO Cellular Location) in regulated (log2 ratio >1.0) non-SQ sites from the Mec1 experiment.
C. String analysis of proteins with Mec1-dependent phosphorylation in non-S/T-Q consensus,

SUPPLEMENTAL FIGURE 4
# Corresponding to Figure 6

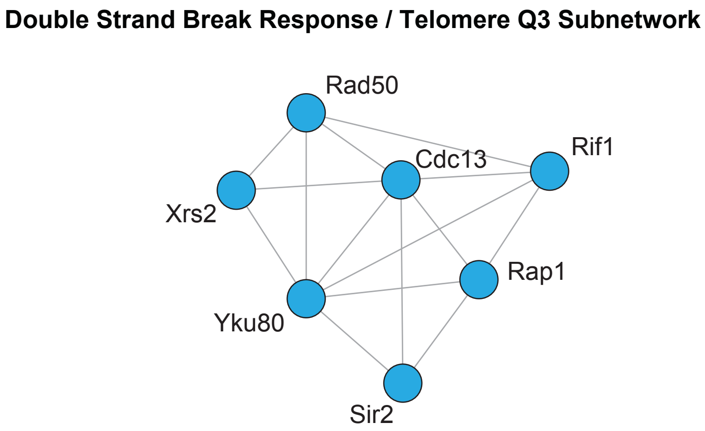

Supplemental Figure S4 (Related to Figure 6).
String analysis of proteins with phosphorylation in Q3 (induced in *rad9Δ mec1Δ* cells) related to telomere maintenance and/or DNA double strand break repair.
